# Commentary: Why Your Body Can Jog Your Mind

**DOI:** 10.3389/fpsyg.2018.00033

**Published:** 2018-02-08

**Authors:** Francesca Pistoia, Marco Sarà, Antonio Carolei, Simona Sacco

**Affiliations:** ^1^Department of Biotechnological and Applied Clinical Sciences, Neurological Institute, University of L'Aquila, L'Aquila, Italy; ^2^Post-Coma Rehabilitative Care Unit, San Raffaele Hospital, Cassino, Italy

**Keywords:** physical exercise, embodied cognition, embodiment, rehabilitation, locked-in syndrome, vegetative state

We read with interest the recently published paper by Macedonia and Repetto that highlights the role of physical activity and gesture use in improving cognitive functions, learning and well-being in healthy subjects (Macedonia and Repetto, 2017). The aim of the authors is to translate recent scientific findings about this issue from neuroscience to the educational and learning framework in schools. It is stressed that better learning may be achieved when conceptual reasoning and thinking are associated with movement, due to the beneficial effects of physical activity on neurogenesis, the modulation of neurotransmitters and neurotrophic factors and neuroplastic brain changes. Benefits arising from physical activity are also evidenced by the observation that gesture use helps to internalize cognitive strategies in everyday performance due to mechanisms of visuo-motor learning facilitation. This occurs not only when gesture use is coupled with cognitive reasoning in the same subject but also when subjects observe others' actions while processing their own cognitive tasks (Conson et al., 2009).

While agreeing with all this evidence, we would like to highlight the educational role that physical activity has not only in healthy subjects but also in patients with a wide spectrum of motor, perceptive, and cognitive dysfunctions as a result of specific neurological diseases. For instance, physical activity has been shown to have protective effects on cognitive functions and memory in patients with Alzheimer's disease (AD), in whom functional alterations of several neurotransmitter systems in the early phases of the disease are driven by the Beta-amyloid action (Mura et al., 2010). The beneficial effect of physical activity would be mediated by the induction of structural and neurochemical changes in the hippocampus and related medial temporal lobe areas that are important for learning and memory (Duzel et al., 2016). Exercise has therefore been considered as a treatment for pre-clinical AD, later stage AD, and as a prevention strategy (Cass, 2017). Similarly, physical activity has been reported to be beneficial in patients with Parkinson's disease (PD), the prevalence of which is progressively increasing as a result of population aging (Totaro et al., 2005). Patients with PD, especially in the early stages of the disease, can benefit from sustained strength training, aerobic training, tai chi, or dance therapy lasting at least 12 weeks (Mak et al., 2017). Some evidence has also demonstrated that physical exercise is able to boost motor learning in patients with PD (Marinelli et al., 2017), especially when the exercise has appropriate intensity and repetition, difficulty, complexity, and specificity (Mehrholz et al., 2015). Moreover, the effectiveness of physical re-education in patients with PD may be improved by combining sensory stimulation, cueing and music in pleasant social contexts and environments that increase task enjoyment (Volpe et al., 2013). Finally, some patients showing the most severe forms of cognitive and motor disability, such as vegetative state (VS) and Locked-In Syndrome (LIS), seem to benefit from rehabilitative approaches based on gesture observation and simulation. For example, a subgroup of patients with VS was recently reported to recover some elementary purposeful motor behaviors when they were stimulated through a combination of Transcranial Magnetic Stimulation and gesture observation (Pistoia et al., 2013b). This suggests that gesture observation may favor the transformation of some perceived actions into motor images and performances, probably depending on the activation of mirror motor neurons and ultimately resulting in the recovery of elementary motor activities in some patients (Sarà and Pistoia, 2010; Pistoia et al., 2013b). Similarly, action observation may improve motor imagery and performance in healthy subjects as well as in patients with the most severe forms of motor disability, including LIS (Conson et al., 2009). LIS is a severe neurological condition characterized by the loss of any motor outputs with the exception of vertical eye movements and blinking. Patients with LIS are conscious but show a dramatic reduction of the motor repertoire, with quadriplegia, anarthria, and lower cranial nerve paralysis. They learn to interact with the environment using eye-coded communication. Moreover, recent evidence suggests that they also show a functional impairment of cortical neuronal synchronization mechanisms (Babiloni et al., 2010) and suffer from non-motor cognitive symptoms that can be interpreted as signs of an embodiment disorder (Sacco et al., 2008; Conson et al., 2010; Pistoia et al., 2010, 2017). Rehabilitative approaches based on the observation of motor performances by others may help patients to overcome their selective motor imagery defects and to promote a virtuous cycle of movement observation, planning, and execution.

The aforementioned syndromes are not the only neurological diseases that can benefit from physical activity: aerobic exercise has been shown to be effective in the treatment of chronic tension-type headache and migraine (Pistoia et al., 2013a; Daenen et al., 2015; Sacco et al., 2015) as well as in the reduction of risk factors associated with stroke (Niewada and Michel, 2016) or affecting stroke's outcomes (Sacco et al., 2007; Mairbäurl, 2013) and in the rehabilitative management of multiple sclerosis (Devasahayam et al., 2017).

In conclusion, physical activity is a valuable educational tool to improve overall brain health not only in healthy subjects but also in patients with various neurological conditions, where neural plasticity may be enhanced either by the physical performance itself or by mechanisms of visuo-motor learning facilitation. It is no accident that the rehabilitative process is defined as an educational process where the patient is asked to relearn previously acquired motor and cognitive skills. For this process to be successful the patient has to learn how to learn. He should be guided in discovering the most effective strategies to recover learning abilities and original skills.

## Author contributions

All authors listed have made a substantial, direct and intellectual contribution to the work, and approved it for publication.

### Conflict of interest statement

The authors declare that the research was conducted in the absence of any commercial or financial relationships that could be construed as a potential conflict of interest.
